# Never Too Soon: An Unusual Case of Intrahepatic Cholestasis of Pregnancy at Five Weeks Gestation

**DOI:** 10.7759/cureus.10540

**Published:** 2020-09-19

**Authors:** Nicha Wongjarupong, Sheila Bharmal, Nicholas Lim

**Affiliations:** 1 Internal Medicine, University of Minnesota, Minneapolis, USA; 2 Division of Gastroenterology, Hepatology and Nutrition, University of Minnesota, Minneapolis, USA

**Keywords:** obstetric cholestasis, liver disease of pregnancy

## Abstract

Intrahepatic cholestasis of pregnancy (ICP) is an incompletely understood liver disease which results in systemic accumulation of bile acids, associated with maternal pruritus and increased risk of intrauterine fetal death. Onset is typically in the third trimester; however, rare cases have been reported early in the first trimester. We present a case of severe, early onset ICP at five weeks gestation in a spontaneous pregnancy. The patient was treated successfully with ursodeoxycholic acid and, after close obstetrical surveillance, delivered a healthy female infant via induced delivery at 34 weeks six days.

## Introduction

Intrahepatic cholestasis of pregnancy (ICP) is a liver disease of undefined etiology and pathogenesis, typically associated with maternal pruritus involving the palms and soles, combined with elevated serum bile acid levels of ≥10 μmol/L.

In general, the maternal course of ICP is benign, with resolution of clinical symptoms and biochemical abnormalities after delivery. Conversely, ICP has more serious consequences for the fetus including increased risk of spontaneous pre-term delivery, lower birth weight, cardiac arrhythmias, meconium-staining of amniotic fluid, infant respiratory distress syndrome, and sudden perinatal death [1-3]. Severe ICP is defined as serum bile acids of more than 40 μmol/L. Severe cholestasis is seen in approximately one-fifth of all cases of ICP and is associated with worse fetal outcomes [1,4,5].

ICP typically occurs during the late second to third trimester of pregnancy. Genetic defects in bile canaliculi transporters such as ATP8B1, ABCB11, ABCB4, ABCC2, NR1H4, and FGF19 can be found in patients with ICP [6]. Estrogen is postulated to play an important role in the onset of ICP given the association of ICP with multi-parity, ovarian hyper-stimulation and prior use of oral contraceptives [7]. ICP typically presents during late pregnancy as estrogen levels start to rise at six to eight weeks gestation and peak at 36 weeks [8]. In the literature review, we found a total of 10 cases of severe ICP during the first trimester reported previously in the literature, of which, our case report at five weeks gestation is the earliest onset of ICP thus far [8-14].

We present a case of severe ICP with symptom onset as early as five weeks gestation prior to obstetric confirmation of pregnancy. Our aim is to raise awareness of this rare presentation of ICP during the early pregnancy, as it is a treatable condition with a favorable outcome if detected.

## Case presentation

A 32-year-old, otherwise healthy gravida 2 para 1, white female at approximately five weeks gestation presented to a local emergency department for pruritus predominantly involving her extremities. At the time, the patient and treating physician were not aware of her pregnancy status. Physical exam was unremarkable and no further testing was pursued. The patient was discharged with a presumed allergic reaction. She completed a short course of prednisone and diphenhydramine without improvement, prompting further visits to the emergency department for similar symptoms.

**Figure 1:**
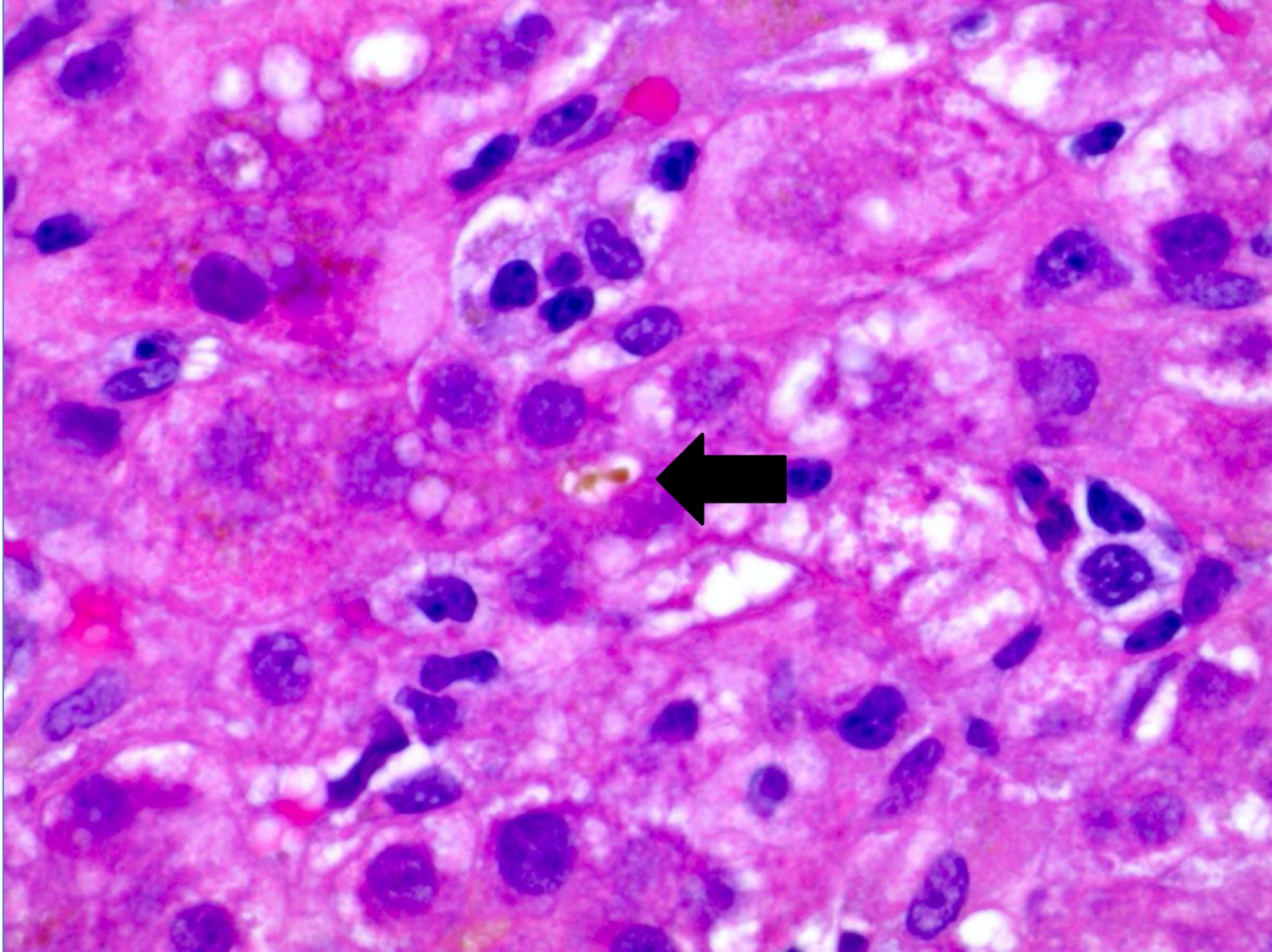
Liver biopsy with canalicular cholestasis (at arrow), H&E, 1000x

**Figure 2:**
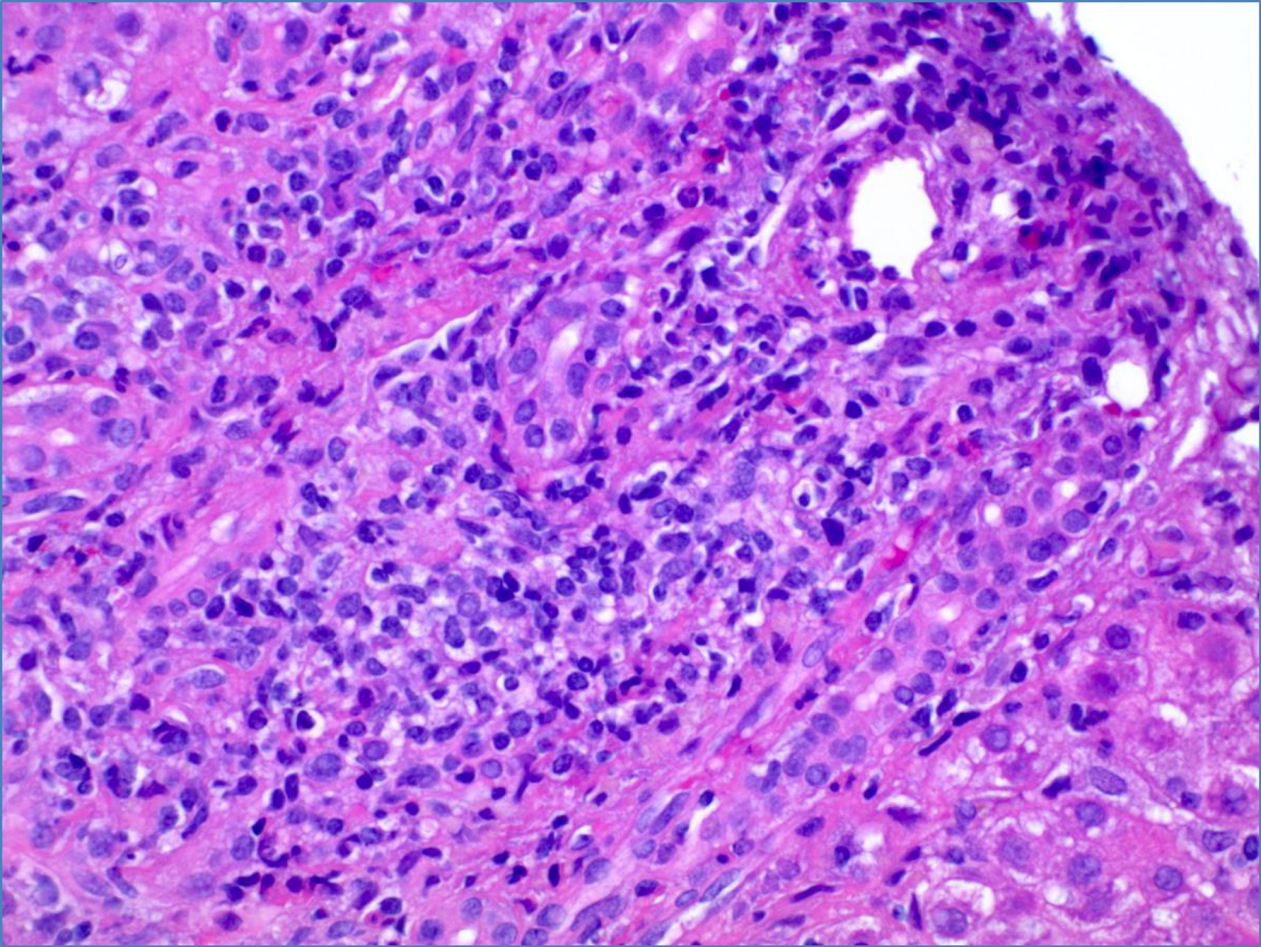
Liver biopsy with mononuclear inflammation and intact bile duct, H&E, 400x

Approximately four weeks after initial presentation, the patient developed nausea and epigastric pain. Urine pregnancy testing was positive. Obstetrical ultrasound confirmed a nine-week gestation monofetal pregnancy. Laboratory testing revealed a total bilirubin 6.1 mg/dL (0.2-1.2 mg/dL), direct bilirubin 4.5 mg/dL (0.1-0.5), indirect bilirubin 1.6 mg/dL (0.2-0.8), alkaline phosphatase 179 IU/L (50-136 IU/L), aspartate aminotransferase 1117 IU/L (2-40 IU/L), alanine aminotransferase (ALT) 1406 IU/L (8-45 IU/L) and international normalized ratio 1 (0.86-1.14). Abdominal ultrasound showed mild diffuse gallbladder wall thickening without cholelithiasis. The common bile duct was normal in caliber and Doppler evaluation showed patent vasculature. Subsequent magnetic resonance cholangiopancreatography was also normal. The patient denied alcohol, acetaminophen or any other toxin use. Extensive laboratory evaluation including viral hepatitis testing (A, B, C, E, Epstein-Barr virus, cytomegalovirus, herpes simplex virus) and autoimmune serologies (ANA, anti-smooth muscle antibody, F-actin antibody IgG, serum immunoglobulin levels, anticardiolipin, beta-2 glycoprotein, SS-A antibody, SS-B antibody, Jo-1 antibody) were negative. Serum ceruloplasmin and alpha-1-antitrypsin were normal. Total bile acids later returned at 462 μmol/L supporting a diagnosis of severe ICP. Liver biopsy was pursued given the patient’s atypical presentation. Histopathology showed mild to moderately severe non-specific lobular hepatitis without evidence of chronic liver disease (Figures 1, 2).

The patient was treated for ICP with ursodeoxycholic acid (UDCA) at 600 mg twice daily with gradual improvement in her pruritus and normalization of liver function tests late in her second trimester. Total bile acid level was monitored periodically and also progressively improved until the time of delivery.

After close obstetrical and biochemical surveillance of the maternal and fetal conditions, the patient was induced at 34 weeks 6 days gestation. Dexamethasone was administered to accelerate fetal lung maturation. The patient delivered a healthy female infant weighing 5 pounds 15 ounces via uncomplicated vaginal delivery. Both patient and baby followed a routine post-partum course with full resolution of symptoms and liver biochemical abnormalities.

## Discussion

ICP is the most common pregnancy-associated liver disease, with a variable incidence worldwide between 0.2% and 25% [15]. The pathophysiology of ICP is incompletely understood. Family clustering and varying incidence in different geographic regions suggest an underlying genetic predisposition. Moreover, genetic defects in at least six canalicular transporters have been identified as associated with ICP [6]. Our patient did not have any obvious risk factors for ICP, and genetic testing was not pursued given that this was her first presentation with ICP.

A diagnosis of ICP should be considered in pregnant patients with intense pruritus of the extremities, concentrated primarily on the hands and soles, in the absence of rash. As the disease progresses, secondary skin changes can range from excoriation to prurigo nodules [16]. Serum bile acid level exceeding 10 mol/L is the gold standard for diagnosis. Higher bile acid levels (>40 μmol/L) are associated with increased rates of spontaneous preterm delivery and asphyxia events [1,4,5]. Biochemical abnormalities are variable and mostly resolve completely after delivery. Serum aminotransferases are usually less than two times the upper limit of normal but may reach values greater than 1000 IU/L. Bilirubin is increased only in exceptional cases [6].

ICP rarely occurs during the first trimester. Our patient first developed pruritus at five weeks gestation and is the earliest reported case of ICP from a spontaneous pregnancy [8]. The onset of ICP in the second and third trimesters is hypothesized to be related to the peak estrogen levels that occur during the later stages of pregnancy [7,8]. Patients who present in the first trimester potentially could have other factors causing elevated estrogen. Our patient did not have any of the defined risk factors for hyperestrogenemia. Of the previous reported cases of first trimester severe ICP, four out of 10 patients underwent ovarian stimulation for in vitro fertilization (IVF) and one patient had a twin pregnancy [8,13]. In women undergoing IVF, additional follicle-stimulating hormone induces an estrogen surge to stimulate ovulation [17]. Only one patient underwent genetic testing which showed homozygous MDR3, and homozygous BSEP mutation, both of which were previously identified in the ICP cohort [10].

On review of 10 cases with first trimester severe ICP, gestational age ranged from five to 11 weeks and maternal age ranged from 21 to 32 years [8-14]. Regarding outcomes, there were no cases of maternal death. There were five patients who had term deliveries, two patients who had preterm delivery, one patient underwent pregnancy termination at 11 weeks due to persistent elevated liver enzymes despite UDCA treatment, one patient who had a blighted ovum, and one patient who developed a spontaneous miscarriage.

Management of ICP is focused on close obstetrical, clinical and biochemical surveillance. Treatment with UDCA improves both transport and secretion of bile acids, minimizing fetal exposure. UDCA is effective in reducing pruritus and improving liver biochemical abnormalities in patients with ICP. It also lowers the rate of prematurity and decreases the need for neonatal intensive care unit admission [18]. Moreover, UDCA is safe and well-tolerated [19]. Earlier delivery is indicated in patients with intractable pruritus, persistence of hepatic dysfunction and past history of bad neonatal outcome due to ICP. The ideal gestational age for elective induction of labor to minimize the risk of perinatal mortality is unknown, but a large cohort study has suggested that delivery at 36 weeks gestation may be optimal [20].

## Conclusions

In conclusion, we present an unusual case of severe, early onset ICP at five weeks gestation prior to obstetric confirmation of pregnancy. ICP remains an enigmatic disease with variable clinical presentations and potentially serious fetal outcomes. Other types of liver disease should always be excluded and treatment with UDCA results in resolution of the symptoms and elevated liver enzymes in the majority of cases. In any female patient of childbearing age presenting with generalized pruritus, a diagnosis of ICP should always be considered regardless of known pregnancy status or the trimester of pregnancy.
